# Factors associated with COVID-19 preventive health behaviors among the general public in Mexico City and the State of Mexico

**DOI:** 10.1371/journal.pone.0254435

**Published:** 2021-07-23

**Authors:** Rosalinda Sánchez-Arenas, Svetlana V. Doubova, Marco Antonio González-Pérez, Ricardo Pérez-Cuevas

**Affiliations:** 1 Epidemiology and Health Services Research Unit CMN Siglo XXI, Mexican Institute of Social Security, Mexico City, Mexico; 2 Facultad Estudios Superiores Iztacala, Universidad Nacional Autónoma de México, Ciudad de México, México; 3 Division of Social Protection and Health, Jamaica Country Office, Interamerican Development Bank, Kingston, Jamaica; The Chinese University of Hong Kong, HONG KONG

## Abstract

**Objective:**

To evaluate factors associated with COVID-19 preventive health behaviors among adults in Mexico City and the State of Mexico.

**Methods and findings:**

We conducted a cross-sectional survey from June to October 2020 through a structured, internet-based questionnaire in a non-probabilistic sample of adults >18 years living in Mexico City and the State of Mexico. The independent variables included sociodemographic and clinical factors; health literacy; access to COVID-19 information; and perception of COVID-19 risk and of preventive measures’ effectiveness. The dependent variable was COVID-19 preventive health behaviors, defined as the number of preventive actions adopted by participants. The data were analyzed through multivariate negative binomial regression analysis. The survey was completed by 1,030 participants. Most participants were women (70.7%), had a high school or above level of education (98.8%), and had adequate health literacy and access to COVID-19 information. Only 18% perceived having a high susceptibility to COVID-19, though 83.8% recognized the disease’s severity and 87.1% the effectiveness of preventive measures. The median number of COVID-19 preventive actions was 13.5 (range 0–19). The factors associated with preventive health behavior were being female, of older age, a professional worker, a homemaker, or a retiree; engaging in regular physical exercise; having high health literacy and access to COVID-19 information sources; and perceiving COVID-19 as severe and preventive measures as effective.

**Conclusion:**

People with high education and internet access in Mexico City and the State of Mexico reported significant engagement in COVID-19 preventive actions during the first wave of the COVID-19 pandemic.

## Introduction

Preventive health behaviors by individuals and communities are the primary strategy for reducing transmission and controlling the spread of any novel aggressive infection that lacks evidence-based treatment and vaccine prevention [1]. Sociodemographic, psychological, information-related, and cultural factors influence individual- and community-level health behaviors [2, 3]. Sociodemographic factors, such as being a woman and being older and more educated increase the probability of preventive health behaviors during a pandemic. Psychological factors also play a role, including perceiving personal susceptibility to a disease and infection severity and trusting the effectiveness of prevention recommendations [3]. Access to health-related information and the ability to understand it, including through risk communication, is also important [4]. Cultural norms also influence health behaviors; countries with strict social norms and penalizations for deviance, such as China and Japan, have been more likely to impose individual and community preventive health behaviors than countries with more permissive social norms, such as Italy and Brazil [5]. Several studies confirmed most of these factors during the early stages of the COVID-19 pandemic [6–11].

There is wide variability in perception of risk and adherence to COVID-19 preventive behaviors within and among countries [10, 11], which, in turn, has influenced the incidence of COVID-19 cases and deaths. The public’s cooperation with prevention efforts has been critical in some countries. For example, with 97 million inhabitants, Vietnam had 1,520 cases and 35 deaths as of January 13, 2021 [12]. This success is in part due to a strong risk communication strategy and the public’s mobilization. Conversely, with 51 million inhabitants, Colombia had 1.8 million cases and 47,124 deaths (adjusted case fatality rate: 2.6) as of January 13, 2021 despite a series of government response measures. Such variability justifies investigating country-specific factors associated with the adoption of preventive health behaviors.

Since March 2020, the COVID-19 pandemic has been imposing a heavy toll on Mexico’s health sector, the economy, and its 129 million inhabitants. As of January 13, 2021, there were 1.5 million cases and 135,680 deaths (adjusted case fatality rate: 10.4); the country also had the lowest number of COVID-19 tests performed in Latin America. Mexico City and the State of Mexico (>30 million inhabitants) have experienced the highest number of COVID-19 cases and deaths. At the onset of the pandemic, the government informed the public about the risks and promoted preventive health behaviors—such as hand sanitizer use, physical distancing, and staying at home—but it also sent contradictory messages. For example, it disregarded the usefulness of face masks. Although some public health actions were enforced, such as closing schools, shopping centers, and churches, and canceling sports events, there were no lockdowns or curfews enforced in high incidence areas. This decision is related to the fact that most Mexicans work in the informal economy and could not stay at home without any income; as this population did not receive monetary support from the government in response to the pandemic, contributing to the ongoing conflict between the need for physical distancing measures and the need to allow people to carry on with their income-generating activities.

Information on the prevalence and factors associated with COVID-19 preventive health behaviors in Mexico is scarce. A study evaluating older adults’ decision to stay at home as a preventive action found that half of the participants perceived a very low or low personal susceptibility to COVID-19. Additionally, higher income and educational attainment increased the probability of staying at home [13]. However, these findings might not be pertinent to other age groups and it is not well established if other factors (e.g., health literacy) are associated with preventive health behaviors, particularly in COVID-19 high incidence areas, such as Mexico City and the State of Mexico. Therefore, the objective of this study was to identify factors associated with COVID-19 preventive health behaviors among adults living in Mexico City and the State of Mexico.

## Materials and methods

We conducted an internet-based mixed-device cross-sectional open survey from June 2020 to October 2020, during the first wave of the COVID-19 pandemic in Mexico.

### Study population

The study included a non-probabilistic sample of adults >18 years from Mexico City and the State of Mexico. The invitation to participate in the survey was disseminated by the Directorate of Economic and Social Benefits of the Mexican Institute of Social Security (IMSS) and by researchers through Twitter, Facebook, and QuestionPro.

### Ethics

Participants received access to the survey after signing an electronic informed consent form. The consent form described the purpose of the study, the content of the survey questionnaire, the approximate length of time to complete it (25–35 minutes); the voluntarily and anonymously nature of the survey (no personal information was collected); the name and contact information of the principal researcher, and the IMSS Ethics Committee. The consent form also specified that the participants could end the survey at any time and that there was no monetary or another type of incentive for participation. The study protocol was approved by the Research and Ethics Committees of the Mexican Institute of Social Security (Register number: R-2020-785-069).

### Study questionnaire

We designed the structured questionnaire following the recommendations of Cummings et al. (1980) [2], Bish and Michie (2010) [3], and Weston and Amlôt (2020) [14] who suggest that the research framework on preventive health behaviors should incorporate a full range of relevant theories and constructs. In keeping with the Protection Motivation Theory, we included questions to measure cognitive and psychological behavioral constructs [15], such as perceived severity of COVID-19, perceived probability of contagion, recommended preventive measures’ effectiveness and self-efficacy. The questionnaire collected participants’ sociodemographic and clinical characteristics, as well as information about their lifestyle, health literacy, access to COVID-19 information, and preventive actions; it included the European Health Literacy Questionnaire and the Short Form Health Survey (SF-12). Three researchers with experience in health services and behavior research assessed the questionnaire’s face validity and reviewed the questions and answer choices [16]. We also pre-tested the questionnaire with 15 adults from diverse educational and occupational backgrounds to ensure that the questions were understandable.

The electronic questionnaire was built using QuestionPro software. The questionnaire allowed an automatic capturing of responses. Cookies were used to assign a unique user identifier. The survey was available on a mobile device, personal computer, laptop, or tablet and was displayed as a continuous run of questions. The completeness check of responses was performed by the researchers after each questionnaire has been submitted. In addition, we eliminated duplicated observations from the same IP addresses (~0.9%), keeping the first complete entry for analysis.

### Variables

The study dependent variable was preventive health behaviors as indicated by the number of COVID-19 preventive actions adopted by participants. We assessed this variable through the question, “What preventive actions do you currently use to avoid contagion or spread of COVID-19?” The question had a list of 17 activities and the response options were “yes” or “no” for each action and one open-ended question about other preventative activities. The list of preventive behaviors included: 1) Frequent washing of hands; 2) Using hand sanitizers; 3) Wearing a face mask when going out; 4) Covering one’s mouth with a sleeve when coughing; 5) Not shaking hands or kissing; 6) Not touching one’s face, eyes, nose, and mouth; 7) Avoiding contact with people with acute respiratory illness; 8) Not meeting with groups of more than five people; 9) Staying at home as much as possible; 10) Working or studying from home; 11) Not going to public places (e.g., shopping malls, cinemas, restaurants); 12) Not traveling; 13) Not using public transportation; 14) Maintaining a physical distance of at least 1.5 meters from others in public areas; 15) Changing and washing clothes after returning home; 16) Disinfecting surfaces; and 17) Assessing COVID-19 risk using government and health institution web applications. We used the sum of the responses to construct the dependent variable. The rational to define the dependent variable as the sum of the preventive measures that participants reported to perform was based on the World Health Organization (WHO) and the Mexican government’s recommendations on preventive measures that should be implemented by the public. Before COVID-19 vaccine was developed, both WHO and the Mexican government, recommended simultaneous realization of multiple complementary preventive actions. For instance, the WHO, on its webpage on “Coronavirus disease (COVID-19) advice for the public”, calls “Do it all!” pointing that to prevent COVID-19, one should adhere to all these measures (https://www.who.int/emergencies/diseases/novel-coronavirus-2019/advice-for-public). Therefore, considering the complementary nature of the studied preventive behaviors, the sum of these behaviors could be an important indicator of the adherence of the population to COVID-19 prevention. This decision is also justifiable in the absence of evidence on the higher effectiveness of one preventive measure compared to the others, or on the standard for each preventive measure.

The independent variables were:

1. Sociodemographic characteristics, including sex; age; residence in Mexico City or the State of Mexico; schooling (secondary school or less, high school with or without technical training, or university); occupation (student, homemaker, unskilled worker, clerk, service and sales worker, health professional, other professional worker, or pensioner/retiree); household composition (living with a life partner, child(ren), or older adult(s)); and regular healthcare provider (social security, Ministry of Health, or private providers).
2. Clinical characteristics, including underlying medical conditions, history of COVID-19 infection, and health-related quality of life. To identify participants with medical conditions that increased their risk of a severe COVID-19 infection, we asked about the presence of one or more of the following conditions [17]: obesity, diabetes, hypertension, cerebrovascular disease, chronic obstructive pulmonary disease, asthma, chronic kidney disease, and cancer. The Mexican version of the Short Form Health Survey (SF-12) comprised of 12 items, measured participants’ quality of life. Physical and mental scores were calculated using an algorithm to convert each item response into standardized values according to specific predetermined weights [18]. Summary scores for each component range from 0–100 and are interpreted as low QoL (close to 0) and high QoL (approaching 100) [19].
3. General health-related behaviors encompass current smoking habits and regular physical exercise (at least 30 minutes five times a week).
4. Health literacy and access to COVID-19 related information. We used the Spanish version of the European Health Literacy Questionnaire (HLS-EU-Q47) to evaluate health literacy related to disease prevention [20]. Fifteen questions constitute this domain, with each measured on a five-point Likert scale (very easy; easy; difficult; very difficult; do not know). We used the formula (mean-1)*50/3 to obtain a health literacy score, with a minimum of 0 (low health literacy) and a maximum of 50 points (high health literacy). We ascertained access to COVID-19 related information by asking participants about the sources of information they used to access or receive information on COVID-19.
5. Psychological factors comprise the perception of COVID-19 risk (susceptibility and severity), the effectiveness of preventive measures and self-efficacy. Perceived susceptibility to COVID-19 was measured by asking the participants, “How likely do you think you are to get infected with COVID-19?” Responses of “very unlikely,” “unlikely,” or “more or less likely” were defined as low/regular susceptibility, while “very likely” and “extremely likely” responses were defined as high susceptibility. We measured the perceived severity of COVID-19 with the question, “How serious do you consider coronavirus infection (COVID-19) to be?” “nothing serious,” “a little bit serious,” and ““somewhat serious”” were defined as responses indicating a low/regular perception of severity, whereas “very serious” and “extremely serious” were considered high severity. The perceived effectiveness of preventive actions was assessed by the question, “How effective do you think the COVID-19 preventive measures recommended by the government are?” “Not at all,” “a little,” or “somewhat” were defined as indicating a perception of low/regular effectiveness; “very much” and “much” were defined as a perception of high effectiveness [1]. The self-efficacy was measured by the question, “How confident are you that you could decrease your chance of COVID-19 infection by performing preventive measures?” “Not at all,” “a little bit,” or “somewhat confident” were defined as indicating a perception of low/regular self-efficacy; “very” and “extremely confident” were defined as a perception of high self-efficacy [21].

### Sample size and statistical analysis

The sample size was based on the practice of ensuring at least ten participants per covariate included in the multiple regression analysis [22].

We performed descriptive and bivariate analyses. The descriptive analysis showed that 22.9% of participants had missing data on one or more of the study variables. To identify if the missing data mechanism was at random or not, to define the strategy to handle missing data, we compared the characteristics of participants with and without missing data using the chi-square test for categorical variables and Student t-tests for continuous variables. This strategy also allowed to describe the differences between these two types of participants.

Moreover, we performed the Kruskal Wallis test to compare medians of the number of COVID-19 preventive activities among different participant subgroups according to their characteristics (e.g., female vs. male). Spearman’s rank correlation test was used to identify the correlation between independent numeric variables (health literacy score, number of sources used to access COVID-19 related information, and physical and mental scores of SF-12) and the dependent variable (number of COVID-19 preventive measures adopted by participants).

As recommended by Van der Weele [23], we performed a multivariate regression analysis with simultaneous inclusion of all conceptually and clinically relevant variables to determine the association between independent variables and the dependent variable. We built a multivariate negative binomial regression model to consider the count nature and skewed distribution of the dependent variable. The coefficients in the multivariate negative binomial regression represent adjusted prevalence ratios (aPR); their interpretation is the same as risk ratios.

We corrected the analysis by stabilized inverse probability weights (IP-weights) to avoid selection bias because of missing data, as we found differences between participants with and without missing data. The IP-weights method is based on assigning a specific weight to each subject with complete information; the subject in the analysis accounts for himself or herself and participants with the same value of covariates who have missing data. This method allows for achieving conditional exchangeability (within the level of measured covariates) of those selected and those who were not selected [24]. Additionally, stabilized weights typically result in narrower 95% confidence intervals (CI) than non-stabilized weights. The denominator for stabilized inverse probability weights was the probability of “having missing data” given the available covariates without missing data. The covariates were the participants’ sex, age, education level, type of regular healthcare provider, household composition, and residence. The numerator was the probability of “having missing data” regardless of the covariates.

We tested but did not find the following interactions: (1) level of education##health literacy; (2) health literacy##access to COVID-19 related information; (3) perceived susceptibility##perceived severity; (4) perceived susceptibility##perceived effectiveness; (5) history of getting COVID-19##perceived susceptibility; (6) history of getting COVID-19##perceived severity.

We used Stata 14.0 (Stata Corp, College Station, Texas, United States) for the statistical analysis; p<0.05 was considered statistically significant.

## Results

The study’s electronic consent form was accessed by 1,732 persons; of these, 1,262 (72.9%) signed the form and answered the questionnaire. However, 232 persons that answered the questionnaire did not meet the inclusion criteria because they lived outside Mexico City or the State of Mexico. Hence, 1,030 participants met the inclusion criteria; of these, 794 (77.1%) completed the questionnaire, while 236 (22.9%) had missing data on one or more of the study variables.

Table 1 presents the characteristics of study participants and compares those with and without missing data. The average age was 37.6 years. Most were women (70.7%), lived in Mexico City (74.9%), and/or had completed a university degree (63.7%) or high school (34.1%). Most respondents were either health professionals (11.9%) or other professionals (29.1%). The sample also included students (29.8%) and clerks (13.2%). Less than half of respondents lived with their life partner (35.7%), older adult(s) (28.9%), or children (42.7%); 14.7% of participants were active smokers, while only 34.2% practiced regular physical exercise during the COVID-19 pandemic. Most (83.4%) were covered by social security. Nearly a third of respondents (29.8%) had underlying medical conditions that increased the risk of COVID-19 severity and 5.2% had contracted and recovered from COVID-19. On a 100-point scale, participants’ average quality of life score was 53.4 in the physical component and 43.4 in the mental component.

**Table 1:** Characteristics of participants with and without missing data.

Variable	Total n = 1030%	Without missing data n = 794%	With missing data n = 236%	P value
**Socio-demographic characteristics**				
**Age**, in years; mean (SD^a^)	37.6 (14.8)	36.7 (14.6)	40.8 (15.2)	0.0001
**Sex**, Female	70.7	69.9	73.3	0.313
**Residence**				
Mexico City	74.9	73.7	78.8	0.110
State of Mexico	25.1	26.3	21.2	
**Education**				
Secondary school or less	2.2	1.6	4.2	0.053
High school with or without technical training	34.1	33.9	34.8	
University	63.7	64.5	61.0	
**Occupation**	n = 1025		n = 231	
Student	29.8	32.6	20.3	0.0001
Professional worker	29.1	29.6	27.3	
Health professional	11.9	11.8	12.1	
Clerk	13.2	13.1	13.4	
Homemaker	6.6	4.9	12.5	
Service and sales worker	3.5	3.2	4.8	
Unskilled worker	2.0	1.3	4.3	
Pensioner or retiree	3.9	3.5	5.2	
**Household composition** **Living with**:				
Life partner	35.7	33.9	41.9	0.023
Child(ren)	42.7	40.9	48.5	0.039
Older adult(s)	28.9	28.1	31.8	0.272
**Regular healthcare provider**				
Social Security	83.4	83.6	82.6	0.935
Ministry of Health	6.0	5.9	6.4	
Private	10.6	10.5	11.0	
**Medical history and quality of life**				
Presence of underlying medical conditions that increase risk of severe illness from COVID-19				
Yes	29.8	30.2	28.4	0.588
History of COVID-19	n = 978		n = 184	
Yes	5.2	5.7	3.3	0.186
Health related quality of life	n = 840		n = 46	
Physical component, mean (SD^a^)	53.4 (6.9)	53.4 (7.0)	53.6 (6.2)	0.8579
Mental component, mean (SD^a^)	43.4 (12.5)	43.5 (12.6)	42.5 (12.2)	0.6062
**General health-related behaviors**				
Smoking	n = 1022		n = 228	
Yes	14.7	14.9	14.0	0.756
Regular physical exercise during COVID-19	n = 1030		n = 236	
Yes	34.2	39.0	17.8	0.0001
**Health literacy and access to COVID19 related information**	n = 799		n = 5	
Health literacy related to prevention, mean (SD^a^)	37.6 (9.4)	37.6 (9.4)	33.3 (13.2)	0.3084
Access to COVID19 related information	n = 903		n = 109	
Internet sites	96.6	97.0	93.6	0.068
Social media (Facebook, Twitter, WhatsApp, Instagram, etc)	92.9	92.9	92.7	0.913
Ministry of Health daily television reports on COVID-19	87.4	87.0	89.9	0.396
Television	86.7	86.8	86.2	0.877
Press (newspaper/magazine print or electronic)	77.1	78.2	68.8	0.029
Ministry of Health website	75.6	75.7	75.2	0.916
Radio	67.9	69.0	59.6	0.049
IMSS website	65.4	66.1	60.5	0.251
Family members	49.5	60.6	12.3	0.0001
E-mails	43.2	43.8	38.9	0.331
Smartphone-messages	34.4	33.9	38.5	0.338
Number of sources used to access COVID-19 related information, mean (SD^a^)				0.0096
Median (minimum-maximum)	7.8 (2.2)	7.9 (2.2)	7.3 (2.3)	
	8 (0–11)	8 (0–11)	8 (0–11)	
**Perceived risk**	n = 963		n = 169	
High susceptibility to COVID-19	18.0	17.5	20.1	0.422
High **s**everity of COVID-19	83.8	84.3	81.6	0.387
**Perceived benefit and self-efficacy**				
High effectiveness of preventive measures	87.1	89.6	75.7	0.0001
High self-efficacy to decrease chance of COVID-19 infection	61.8	63.3	54.4	0.030

^a^SD: Standard deviation.

On a 50-point scale, the average health literacy score related to prevention was 37.6, considered as sufficient health literacy. Most respondents had access to multiple sources of COVID-19 information, predominantly from the internet (96.6%), social media (92.9%), Ministry of Health daily reports (87.4%), television (86.7%), and the press (77.1%). Regarding the perceived risk of COVID-19, only 18% recognized high personal susceptibility to COVID-19, 83.8% acknowledged the severity of COVID-19, 87.1% the effectiveness of preventive measures and 68.1% reported high perceived self-efficacy to decrease chance of COVID-19 infection.

Participants with missing data were older than those without missing information and were more often homemakers, sales workers, unskilled workers, or pensioners. In addition, compared to those without missing data, participants with missing information were more likely to live with life partners and children, not practice regular physical exercise, have access to fewer sources of COVID-19 information, and perceive a low effectiveness of preventive actions and self-efficacy.

Table 2 shows COVID-19 preventive behaviors adopted by participants. In descending order, 85.9% reported increasing hand washing, 85.4% wearing a face mask when going out, 80.8% covering their mouth with a sleeve when coughing, 80.5% using hand sanitizers, 77.4% avoiding handshakes and kisses, 77.1% avoiding meetings meeting with groups of more than five people, 73.7% avoiding contact with people with acute respiratory illness, 72.7% keeping at least 1.5 meters distance from others in public areas, 71.1% avoiding public places, 68.5% avoiding face touching, 68.2% avoiding travel, 64.7% not using public transportation, 62.1% staying home, 61% working or studying from home, 56.1% disinfecting surfaces, 42.7% changing and washing clothes after being out of the house, 26.7% performing COVID-19 risk assessment through government and health institution applications, and 22.6% other behaviors (e.g., using a face shield or asking younger relatives/friends to do their grocery shopping). The median number of COVID-19 preventive actions undertaken by participants was 13.5, ranging from 0 to 19. Participants with missing information engaged in fewer preventive behaviors than those with complete data.

**Table 2:** COVID-19 preventive actions.

Variable	Total n = 1030%	Without missing data n = 794%	With missing data n = 236%	P value
Frequent washing of hands	85.9	98.2	44.5	0.0001
Wearing a face mask when going out	85.4	98.0	43.2	0.0001
Covering one’s mouth with a sleeve when coughing	80.8	92.4	41.5	0.0001
Using hand sanitizers	80.5	92.1	41.5	0.0001
Not shaking hands or kissing	77.4	88.2	41.1	0.0001
Not meeting with groups of more than five people	77.1	87.7	41.5	0.0001
Avoiding contact with people with acute respiratory illness	73.7	84.0	39.0	0.0001
Maintaining a physical distance of at least 1.5 meters from others in public areas	72.7	83.0	38.1	0.0001
Not going to public places (e.g., shopping malls, cinemas, restaurants)	71.1	81.5	36.0	0.0001
Not traveling	68.2	78.2	34.3	0.0001
Not touching one’s face, eyes, nose, and mouth	68.5	77.8	36.9	0.0001
Not using public transportation	64.7	73.3	35.6	0.0001
Staying at home as much as possible	62.1	70.9	32.6	0.0001
Working or studying from home	61.0	69.9	30.9	0.0001
Disinfecting surfaces	56.1	64.1	29.2	0.0001
Changing and washing clothes after returning home	42.7	49.1	21.2	0.0001
Assessing COVID-19 risk using government and health institution web applications	26.7	30.0	15.7	0.0001
Others (e.g., using face shield, asking younger relatives/friends to do grocery shopping to avoid going out, etc.)	22.6	24.8	15.2	0.002
Number of COVID-19 preventive measures used by participant, mean (standard deviation) median (minimum-maximum)	11.8 (5.3) 13.5 (0–19)	13.5 (3.0) 14 (1–19)	6.2 (7.1) (0–19)	0.0001

Table 3 describes the number of COVID-19 preventive actions adopted by participants according to their characteristics. Compared with their counterparts, female participants, pensioners; and homemakers, and those who lived with older adults, exercised regularly, had high health literacy, accessed more sources of COVID-19 information, and perceived high COVID-19 severity and effectiveness of preventive measures reported undertaking more preventive actions.

**Table 3:** Number of COVID-19 preventive actions used by participants according to their characteristics (n = 794).

Variable	Number of COVID-19 preventive measures		P value
	Mean (SD^a^)	Median (min-max)	
**Socio-demographic characteristics**			
**Sex**			
Female	13.8 (2.8)	14 (1–19)	0.0001
Male	12.8 (3.4)	13 (1–19)	
**Residence**			0.1915
Mexico City	13.5 (3.1)	14 (1–19)	
State of Mexico	13.3 (2.8)	14 (1–19)	
**Education**			0.4707
Secondary school or less	12.5 (3.8)	13 (5–18)	
High school with or without technical training	13.4 (2.8)	14 (4–18)	
University	13.5 (3.1)	14 (1–19)	
**Occupation**			
Student	13.1 (2.9)	14 (4–18)	0.0022
Professional worker	13.7 (3.1)	14 (1–18)	
Health professional	13.8 (2.7)	14 (7–19)	
Clerk	13.4 (3.3)	14 (3–18)	
Homemaker	14.6 (2.5)	15 (9–18)	
Service and sales worker	12.2 (3.6)	14 (6–17)	
Unskilled worker	11.3 (3.6)	11.5 (5–17)	
Pensioner or retiree	14.6 (2.4)	15 (8–18)	
**Household composition Living with**:			
Life partner	13.5 (3.1)	14 (1–19)	0.9385
Without life partner	13.5 (3.0)	14 (2–18)	
Older adult	13.8 (3.1)	14 (3–19)	0.0172
Without older adult	13.3 (3.0)	14 (1–19)	
Child(ren)	13.2 (3.3)	14 (1–19)	0.1644
Without Child(ren)	13.6 (2.8)	14 (2–18)	
**Regular healthcare provider**			0.7426
Social Security	13.5 (3.0)	14 (1–19)	
Ministry of Health	13.2 (3.2)	14 (2–18)	
Private	13.4 (2.8)	14 (6–18)	
**Medical history and quality of life**			
Presence of underlying medical conditions that increase risk of severe illness from COVID-19			0.0608
Yes	13.8 (2.9)	14 (1–18)	
No	13.3 (3.0)	14 (1–19)	
History of COVID-19			0.2260
Yes	14.1 (2.7)	14 (8–19)	
No	13.4 (3.0)	14 (1–19)	
Health related quality of life	Spearman’s rho		
Physical component	-0.027		0.4419
Mental component	0.0358		0.3140
**General health-related behaviors**	Mean (SD^a^)	Median (min-max)	
Smoking			0.8947
Yes	13.5 (3.2)	14 (1–19)	
No	13.5 (3.0)	14 (2–18)	
Regular physical exercise during COVID-19 pandemic			0.0001
Yes	14.0 (3.0)	14 (5–19)	
No	13.1 (3.0)	14 (1–18)	
**Health literacy and access to COVID-19 related information**	Spearman’s rho		
Health literacy related to prevention	0.153		0.00001
Number of sources used to access COVID-19 related information	0.165		0.00001
**Perceived risk**	Mean (SD^a^)	Median (min-max)	
Susceptibility to COVID-19			0.0821
Low/regular	13.6 (3.0)	14 (1–19)	
High	13.1 (3.2)	14 (1–19)	
Severity of COVID-19			0.0004
Low/regular	12.4 (3.5)	13 (1–18)	
High	13.7 (2.9)	14 (1–19)	
**Perceived benefit and self-efficacy**			
Effectiveness of preventive measures			0.0067
Low/regular	12.3 (3.8)	13 (2–18)	
High	13.6 (2.9)	14 (1–19)	
Self-efficacy to decrease chance of COVID-19 infection			0.0729
Low/regular	12.2 (4.8)	13 (0–18)	
High	12.9 (4.1)	14 (0–19)	

^a^SD: Standard deviation

Table 4 depicts the univariate and multivariate negative binomial regression results for factors associated with a higher number of adopted COVID-19 preventive actions. Both analyses showed similar results. The results indicate that being female, older, a health or other professional, a homemaker, and/or a pensioner or retiree; exercising regularly; having a high level of health literacy and access to COVID-19 information sources; and/or perceiving COVID-19 as a high severity infection and preventive measures as highly effective were associated with an increased probability of engaging in a higher number of COVID-19 preventive actions. At the same time, living with a life partner and perceiving a high personal susceptibility to COVID-19 were associated with the adoption of fewer preventive actions.

**Table 4:** Factors associated with the use of COVID-19 preventive actions.

Variable	Crude Prevalence Ratio [95% Conf. Interval], p-value. Corrected by IP-weights	Adjusted Prevalence Ratio [95% Conf. Interval], p-value. Corrected by IP-weights
Observations	n = 794	n = 794
**Socio-demographic characteristics**		
**Sex**		
Female	**1.08 [1.04, 1.13], 0.0001**	**1.07 [1.04, 1.11], 0.0001**
Male	Ref.	Ref.
**Age** in years	**1.002 [1.0008, 1.003], 0.0001**	**1.002 [1.0002, 1.003], 0.029**
**Residence**		
Mexico City	1.01 [0.98, 1.05], 0.416	1.008 [0.98, 1.04], 0.613
State of Mexico		Ref.
**Education**		
Secondary school or less	Ref.	Ref.
High school with or without technical training	1.07 [0.92, 1.24], 0.353	1.04 [0.91, 1.20], 0.520
University	1.08 [0.93, 1.25], 0.316	0.99 [0.87, 1.14], 0.910
**Occupation**		
Student	Ref.	Ref.
Professional worker	**1.04 [1.003, 1.08], 0.033**	**1.09 [1.02, 1.15], 0.006**
Health professional	**1.05 [1.005, 1.11], 0.030**	**1.08 [1.01, 1.15], 0.017**
Clerk	1.02 [0.97, 1.08], 0.400	1.05 [0.99, 1.12], 0.112
Homemaker	**1.11 [1.04, 1.17], 0.001**	**1.10 [1.03, 1.19], 0.008**
Service and sales worker	0.93 [0.82, 1.05], 0.219	1.01 [0.90, 1.12], 0.925
Unskilled worker	0.85 [0.70, 1.03], 0.092	0.86 [0.71, 1.04], 0.124
Pensioner or retiree	**1.12 [1.05, 1.19], 0.001**	**1.10 [1.01, 1.20], 0.026**
**Household composition Living with**:		
Life partner	0.99 [0.96, 1.03], 0.902	**0.96 [0.93, 0.99], 0.048**
Without life partner	Ref	Ref
Older adult	1.04 [1.0008, 1.07], 0.045	1.02 [0.99, 1.05], 0.227
No	Ref.	Ref.
Child(ren)	0.97 [0.94, 1.001], 0.058	0.98 [0.95, 1.01], 0.229
No	Ref.	Ref.
**Regular healthcare provider**		
Social Security	1.02 [0.95, 1.10], 0.564	1.03 [0.96, 1.10], 0.381
Ministry of Health	Ref.	Ref.
Private	1.01 [0.93, 1.10], 0.820	1.04 [0.96, 1.12], 0.317
**General health-related behaviors**		
Smoking		
Yes	0.99 [0.95, 1.04], 0.882	1.004 [0.96,1.05], 0.835
No	Ref.	Ref.
Regular physical exercise during COVID-19		
Yes	**1.07 [1.03, 1.10], 0.0001**	**1.06 [1.03, 1.09], 0.0001**
No	Ref.	Ref.
**Medical history and quality of life**		
Presence of underlying medical conditions that increase risk of severe illness from COVID-19		
Yes	1.03 [0.99, 1.07], 0.057	1.01 [0.98, 1.04], 0.447
No	Ref.	Ref.
History of COVID-19		
Yes	1.05 [0.99, 1.11], 0.100	1.06 [0.99, 1.12], 0.063
No	Ref.	Ref.
Health related quality of life		
Physical component	0.99 [0.99, 1.001], 0.259	0.99 [0.99, 1.002], 0.849
Mental component	1.0002 [0.99, 1.001], 0.780	0.99 [0.99, 1.0003], 0.144
**Health literacy and access to COVID-19 related information**		
Health literacy related to prevention	**1.003 [1.001, 1.004], 0.001**	**1.002 [1.0005, 1.004], 0.012**
Number of sources used to access COVID-19 related information	**1.02 [1.01, 1.03], 0.0001**	**1.02 [1.01, 1.03], 0.0001**
**Perceived risk**		
Susceptibility to COVID-19		
Low/regular	Ref.	Ref.
High	0.97 [0.92, 1.01], 0.158	**0.95 [0.91, 0.99], 0.026**
Severity of COVID-19		
Low/regular	Ref.	Ref.
High	**1.10 [1.04, 1.16], 0.0001**	**1.07 [1.02, 1.12], 0.005**
**Perceived benefit and self-efficacy**		
Effectiveness of preventive measures		
Low/regular	Ref.	Ref.
High	**1.10 [1.03, 1.18], 0.005**	**1.08 [1.01, 1.15], 0.021**
Self-efficacy to decrease chance of COVID-19 infection		
Low/regular	Ref.	Ref.
High	1.02 [0.99, 1.06], 0.147	1.006 [0.97, 1.04], 0.712

The bold values highlight the statistically significant Prevalence Ratios.

## Discussion

This study found a high number of self-reported COVID-19 preventive health behaviors among a study population with a high educational level in Mexico City and the State of Mexico. It also identified that multiple sociodemographic factors, together with health literacy, access to COVID-19 information, perception of the disease’s severity, and prevention benefits increased the likelihood of engaging in more COVID-19 preventive actions.

At the beginning of the COVID-19 pandemic, worldwide reporting indicated a significant uptake of preventive health behaviors, though prevalence of their adoption varied widely. In our study, the most frequently reported behavior was handwashing (85.9%) and the least reported behaviors were changing and washing clothes after returning home (42.7%) and using government and health institution applications for COVID-19 risk assessments (26.7%). In contrast, the most commonly reported preventive measure in Hong Kong, China [1], and South Korea [6] was wearing face masks (over 98%); in Italy, avoiding crowded places (99%) [11]; in Germany, canceling social events (97%) [11]; in the Netherlands, handwashing frequently [11]; and in Iran [7], avoiding handshakes and kisses. A less-frequently reported COVID-19 preventive measure in the Netherlands and Germany was carrying hand gel sanitizer (28.3% and 47%, respectively) [11]; in South Korea, avoiding crowded places (41.5%) [6]; in Italy, keeping children home before any mandates were put in place (60.6%) [11]; and in Iran [7], avoiding face-touching (33.5%). Moreover, the average number of preventive health behaviors reported by the participants in our study was higher than those reported in other countries, such as France (11.8 vs. 6.6 preventive actions, respectively) [9].

Compared to the above-mentioned studies performed primarily in the early stages of the COVID-19 pandemic (January–April 2020), our study was conducted from June to October 2020 when the Mexican government reopened public places and reactivated non-essential services. At the beginning of the study, on June 1, 2020, Mexico had 93,435 confirmed COVID-19 cases and 10,167 confirmed deaths. Information on the pandemic’s origin and magnitude was widely reported both by the Mexican government and internationally. However, in contrast with international recommendations, information on individual COVID-19 preventive actions was limited or controversial. For instance, at the beginning of the pandemic, Mexican health authorities disregarded the effectiveness of face masks for COVID-19 prevention, requiring its use only in supermarkets or other stores; they instead primarily promoted handwashing, hand sanitizer use, physical distancing, and remaining at home.

Health literacy and access to multiple sources of COVID-19 prevention information increased the likelihood that the public would engage in more COVID-19 preventive actions. Health literacy embodies cognitive and social skills and “entails the motivation, knowledge, and competencies to access, understand, appraise and apply health information to make judgments and decisions in everyday life regarding disease prevention and self-care” [20]. Low health literacy skills are associated with higher healthcare expenditures [25]; however, the effect of health literacy on infection prevention, and particularly on COVID-19 preventive health behaviors, is understudied [4]. A recent study from Australia found that people with low health literacy were more likely to hold misinformed beliefs about COVID-19 and vaccinations (in general) than those with adequate health literacy [26]. In addition, a study from China found that health and e-health literacy are significant predictors of preventive health behaviors [27, 28]. In our study, the average health literacy score reflected sufficient health literacy in participants and access to a median of eight sources of information on COVID-19. Moreover, higher health literacy and access to information were associated with engaging in more COVID-19 preventive health actions. Therefore, to improve public adoption of COVID-19 preventive measures, health literacy focused on prevention and access to relevant information sources should be promoted by health professionals and the media.

Similar to other studies [7, 8, 29], perceived severity of COVID-19 was frequent in our population (above 80%). Yet, contrary to studies from Hong Kong [8], where 89% said that they were at risk for COVID-19, or Iran [7], where 70.3% considered themselves susceptible to coronavirus, only 18% of our population perceived high personal susceptibility to COVID-19. The low perception of risk posed by the COVID-19 pandemic was also reported in the US general population [30, 31], where “health messaging about COVID-19 has been extremely confusing, rapidly changing, and politically charged” [32]—a situation that was similar to Mexico. Therefore, it is important that communication by health authorities on COVID-19 prevention is evidence-based and unambiguous.

As found in studies from China, South Korea [2, 6, 8, 33], and the US [31], perceived severity of COVID-19 and effectiveness of preventive actions were associated with higher adoption of COVID-19 preventive activities. Surprisingly, in our study, perception of high susceptibility to COVID-19 was associated with fewer preventive activities. This finding may be explained by the fatalism previously described in Latino populations—a cultural belief that little can be done to change one’s fate, negatively affecting adoption of preventive health behaviors [34, 35]. Exploring and targeting fatalistic beliefs related to COVID-19 through educational mass media interventions can be pertinent to prevention efforts in Mexico.

Several sociodemographic factors were associated with uptake of preventive measures. Similar to other studies, COVID-19 preventive health actions were more frequent in women [6, 7, 36] and increased with age [6, 37]. Professional workers, homemakers, and retirees were associated with higher adoption of preventive actions. This finding highlights the need for active promotion of COVID-19 preventive health behaviors among men, young adults, and those without professional training, as the risk of others (e.g., older adults and children) is dependent on joint preventive efforts and an unwillingness to contribute to the collective good is unfair to other members of society [38].

Finally, physical activity was associated with an increased probability of COVID-19 preventive behaviors. Physical activity is a known protective factor against chronic diseases; recently, several studies identified its association with higher resilience, positive attitude, and decreased depressive symptoms during the COVID-19 pandemic [39, 40]. Our study adds an additional benefit of physical activity given its association with a higher number of COVID-19 preventive actions. This evidence highlights the importance of promoting and facilitating physical activity during the pandemic.

This study has several limitations. First, the information was collected through an online survey disseminated via Twitter and Facebook; thus, it lacked a random selection of study participants since people who do not use these social media platforms or do not have access to the internet were underrepresented. Second, there were few people with low education; only 1.6% of the survey participants had completed secondary school or lower. Although this limits the generalizability of the study results, the 2019 report of the National Institute for the Evaluation of Education (Instituto Nacional para la Evaluación de la Educación, INEE) showed that 53% of the population of Mexico City and 63.4% of the State of Mexico has completed high school [41]. Third, due to the self-reported nature of health behaviors, the results may be prone to social desirability bias. However, responses to online and self-administered questionnaires may be less biased than face-to-face or telephone interviews [42]. Fourth, 22.9% had missing information, which can cause selection bias. To avoid such bias, we corrected the study analysis by stabilized IP-weights. Fifth, we used the Protection Motivation Theory as the study theoretical framework, yet to avoid excessive lengthening of the questionnaire we only measured the main concepts of this theory without measuring such concepts as rewards and response cost. Sixth, this was a cross-sectional study, which does not allow for making causal inferences or identifying the direction of the association between the study variables.

## Conclusion

The general population with a high educational level and access to the internet, Twitter, and/or Facebook in Mexico City and the State of Mexico reported high engagement in COVID-19 preventive actions during the first wave of the COVID-19 pandemic. Greater health literacy, access to information, perception of the disease’s severity and benefits of prevention, as well as sociodemographic factors were associated with a higher number of COVID-19 preventive actions by the studied population. To increase the adoption of COVID-19 preventive behaviors, health communication on this topic should target groups with lower engagement in preventive measures, such as men, younger adults, unskilled workers, and those with low health literacy and a perception of low personal susceptibility to COVID-19 and low severity of the disease.

## Supporting information

S1 File Web questionnaire. Inglish translation.
(DOCX)

S2 File Web questionnaire/cuestionario web. Spanish.
(DOCX)

S3 File Study database. (DTA)

S4 File Checklist for Reporting Results of Internet E-Surveys (CHERRIES). (DOCX)

## Abbreviations

aPR: adjusted prevalence ratios
CI: confidence intervals
HLS-EU-Q47: European Health Literacy questionnaire
IMSS: Mexican Institute of Social Security
IP-weights: inverse probability weights
SF-12: Short Form Health Survey questionnaire
WHO: World Health Organization
