# Supplementary material for: Factors associated with COVID-19 preventive health behaviors among the general public in Mexico City and the State of Mexico
Source: PLoS One. 2021 Jul 23;16(7):e0254435. doi: 10.1371/journal.pone.0254435 (PMC8301657; doi:10.1371/journal.pone.0254435)
Supplement: S1 File — Inglish translation. (DOCX) [file pone.0254435.s001.docx]

**S1** **File. WEB QUESTIONNAIRE-English translation**

Research Project entitled "Risk and protection factors of preventive behaviors of COVID-19"

Answer every question by selecting the response that you consider appropriate and complete the information where requested.

**1. GENERAL INFORMATION**

**1.1. What is your age** (completed years)? _________

**1.2. What is your sex?**

(1) Female

(2) Male

**1.3. Where you live?**

(1) Mexico City

(2) State of Mexico

(3) Other. Specify________________________

**1.4. What health services do you usually use?**

(1) Social security institutions (Instituto Mexicano del Seguro Social (IMSS), or Institute of Security and Social Services of State Workers (ISSSTE), or Petróleos Mexicanos (PEMEX), or Health Institutions of the Ministry of Defense National (SEDENA))

(2) Health facilities of the Ministry of Health / Institute of Health for Wellbeing

(3) Private consultation / private hospital

**1.5. What is your occupation?**

(1) Student

(2) Professional worker (is the person who exercises a profession based on the knowledge acquired in colleges or university)

(3) Health professional

(4) Clerk

(5) Homemaker

(6) Service and sales worker

(7) Unskilled workers (laborer, cleaning, quartermaster, waitress)

(8) Pensioner or retiree

(9) Other. Specify_______________________________

**1.6. What was the highest grade/level of school you completed?**

(1) none

(2) Primary

(3) Secondary

(4) High school or baccalaureate or technical or commercial career

(5) Bachelor's degree

(6) Postgraduate

**1.7. What is your marital status?**

(1) Single

(2) Married

(3) Free union

(4) Divorced

(5) Separated

(6) Widower

**1.8. Do you live with children and adolescents under the age of 18?**

(1) Yes

(2) No

**1.9. Do you live with older adults (65 years and over)?**

(1) Yes

(2) No

**2. HISTORY OF DISEASES**

**2.1. Do you have any chronic diseases?**

(1) Yes

(2) No -> In case of negative answer, go to question 3.1

**2.2. What chronic disease do you have?** (Please, specify all chronic diseases that you have)

| (1). Diabetes  (2). Arterial hypertension  (3). Cardiovascular disease  (4). Chronic kidney disease  (5). Cancer  (6). Obesity  (7). Chronic obstructive pulmonary disease, or asthma  (8) Other | **(1) Yes** | **(0) No** |
| --- | --- | --- |

**2.3 Did you have the COVID-19 disease this year?**

(1) Yes

(2) No

(3) I don't know

**3. HABITS**

**3.1 Do you smoke?**

(1) Yes

(2) No

**3.2 During the pandemic, have you performed physical exercise (e.g. brisk walking, swimming, cycling, etc.) on a regular basis (at least 5 times a week - 30 minutes per session)?**

(1) Yes

(2) No

**4. COVID-19**

**4.1. “How likely do you think you are to get infected with COVID-19?”**

(1) Very unlikely

(2) Unlikely

(3) More or less likely

(4) Very likely

(5) Extremely likely

**4.2 How serious do you consider coronavirus infection (COVID-19) to be?”**

(1) Nothing serious

(2) A little bit serious

(3) Somewhat serious

(4) Very serious

(5) Extremely serious

**4.3 How effective do you think the COVID-19 preventive measures recommended by the government are?**

(1) Very much

(2) Much

(3) Somewhat

(4) A little

(5) Not at all

**4.4 How confident are you that you could decrease your chance of COVID-19 infection by performing preventive measures?**

(1) Not at all

(2) A little bit confident

(3) Somewhat confident

(4) Very confident

(5) Extremely confident

4.5. What sources of information you use to access or receive information on COVID-19 epidemic? (Please, specify all sources that you use)

| Source(s) | (1) Yes | (0) No |
| --- | --- | --- |
| (1) Television |  |  |
| (2) Radio |  |  |
| (3) Press (newspaper/magazine print or electronic) |  |  |
| (4) Internet sites |  |  |
| (5) IMSS website |  |  |
| (6) Ministry of Health website |  |  |
| (7) E-mails |  |  |
| (8) Social media (Facebook, Twitter, WhatsApp, Instagram, etc) |  |  |
| (9) Ministry of Health daily television reports on COVID-19 |  |  |
| (10) Smartphone-messages |  |  |
| (11) Family members |  |  |

4.6 What preventive actions do you currently use to avoid contagion or spread of COVID-19?” (Please, specify all preventive actions that you perform)

|  | **(1) Si** | **(0) No** |
| --- | --- | --- |
| (1) Frequent washing of hands |  |  |
| (2) Using hand sanitizers |  |  |
| (3) Wearing a face mask when going out |  |  |
| (4) Covering one’s mouth with a sleeve when coughing |  |  |
| (5) Not touching one’s face, eyes, nose, and mouth |  |  |
| (6) Avoiding contact with people with acute respiratory illness |  |  |
| (7) Frequent disinfecting of surfaces |  |  |
| (8) Maintaining a physical distance of at least 1.5 meters from others in public areas |  |  |
| (9) Assessing COVID-19 risk using government and health institution web applications |  |  |
| (10) Not shaking hands or kissing |  |  |
| (11) Changing and washing clothes after returning home |  |  |
| (12) Not using public transportation |  |  |
| (13) Not going to public places (e.g., shopping malls, cinemas, restaurants) |  |  |
| (14) Not meeting with groups of more than five people |  |  |
| (15) Working or studying from home |  |  |
| (16) Staying at home as much as possible |  |  |
| (17) Not traveling (outside the city or country) |  |  |
| (18) Asking younger relatives/friends to do grocery shopping to avoid going out |  |  |
| (19) Others (Please, specify)___________________ |  |  |

**5. Health perception (SF-12)**

| Answer every question by selecting the response that you consider appropriate. |  |
| --- | --- |
| 5.1. In general, would you say your health is: | \| 1 \| 2 \| 3 \| 4 \| 5 \| \| --- \| --- \| --- \| --- \| --- \| \| Excellent \| Very Good \| Good \| Fair \| Poor \| |
| The following two questions are about activities you might do during a typical day. Does YOUR HEALTH NOW LIMIT YOU in these activities? If so, how much? | |
| 5.2. MODERATE ACTIVITIES, such as moving a table, pushing a vacuum cleaner, or bowling | \| 1 \| 2 \| 3 \| \| --- \| --- \| --- \| \| Yes, Limited A Lot \| Yes, Limited A Little \| No, Not Limited At All \| |
| 5.3. Climbing SEVERAL flights of stairs: | \| 1 \| 2 \| 3 \| \| --- \| --- \| --- \| \| Yes, Limited A Lot \| Yes, Limited A Little \| No, Not Limited At All \| |
| During the PAST 4 WEEKS have you had any of the following problems with your work or other regular activities AS A RESULT OF YOUR PHYSICAL HEALTH?  5.4. ACCOMPLISHED LESS than you would like:   1. Yes 2. No | |
| 5.5. Were limited in the KIND of work or other activities:   1. Yes 2. No | |
| During the PAST 4 WEEKS, were you limited in the kind of work you do or other regular activities AS A RESULT OF ANY EMOTIONAL PROBLEMS (such as feeling depressed or anxious)?  5.6. ACCOMPLISHED LESS than you would like   1. Yes 2. No | |
| 5.7. Didn’t do work or other activities as CAREFULLY as usual   1. Yes 2. No | |
| 5.8. During the PAST 4 WEEKS, how much did PAIN interfere with your normal work (including both work  outside the home and housework)? | \| 1 \| 2 \| 3 \| 4 \| 5 \| \| --- \| --- \| --- \| --- \| --- \| \| Not At All \| A Little Bit \| Moderately \| Quite A Bit \| Extremely \| |
| The next three questions are about how you feel and how things have been DURING THE PAST 4 WEEKS. For each question, please give the one answer that comes closest to the way you have been feeling. How much of the time during the PAST 4 WEEKS – | |
| \|  \| 1 \| 2 \| 3 \| 4 \| 5 \| 6 \| \| --- \| --- \| --- \| --- \| --- \| --- \| --- \| \|  \| All of the Time \| Most of the Time \| A Good Bit of the Time \| Some of the Time \| A Little of the Time \| None of the Time \| \| 5.9. Have you felt calm and peaceful? \|  \|  \|  \|  \|  \|  \| \| 5.10. Did you have a lot of energy? \|  \|  \|  \|  \|  \|  \| \| 5.11. Have you felt downhearted and blue? \|  \|  \|  \|  \|  \|  \| \| 5.12. During the PAST 4 WEEKS, how much of the time has your PHYSICAL HEALTH OR EMOTIONAL PROBLEMS interfered with your social activities (like visiting with friends, relatives, etc.)? \|  \|  \|  \|  \|  \|  \| | |

| **6. Health literacy (Disease prevention domain** of the **HLS-EU-Q47)** | **Very easy** | **Fairly**  **Easy** | **Fairly**  **Difficult** | **Very difficult** | **Don't know** |
| --- | --- | --- | --- | --- | --- |
| On a scale from very easy to very difficult, how easy would you say it is to: … | 1 | 2 | 3 | 4 | 5 |
| (6.1) find information about how to manage unhealthy behaviour such as smoking, low physical activity and drinking too much? |  |  |  |  |  |
| (6.2) find information on how to manage mental health problems like stress or depression? |  |  |  |  |  |
| (6.3) find information about vaccinations and health screening that you should have? |  |  |  |  |  |
| (6.4) find information on how to prevent or manage conditions like being overweight, high blood pressure or high cholesterol? |  |  |  |  |  |
| (6.5) understand health warnings about behaviour such as smoking, low physical activity and drinking too much? |  |  |  |  |  |
| (6.6) understand why you need vaccinations? |  |  |  |  |  |
| (6.7) understand why you need health screenings? |  |  |  |  |  |
| (6.8) judge how reliable health warnings are, such as smoking, low physical activity and drinking too much? |  |  |  |  |  |
| (6.9) judge when you need to go to a doctor for a check-up? |  |  |  |  |  |
| (6.10) judge which vaccinations you may need? |  |  |  |  |  |
| (6.11) judge which health screenings you should have? |  |  |  |  |  |
| (6.12) judge if the information on health risks in the media is reliable? |  |  |  |  |  |
| (6.13) decide if you should have a flu vaccination? |  |  |  |  |  |
| (6.14) decide how you can protect yourself from illness based on advice from family and friends? |  |  |  |  |  |
| (6.15) decide how you can protect yourself from illness based on information in the media? |  |  |  |  |  |
