# Supplementary material for: Factors associated with COVID-19 preventive health behaviors among the general public in Mexico City and the State of Mexico
Source: PLoS One. 2021 Jul 23;16(7):e0254435. doi: 10.1371/journal.pone.0254435 (PMC8301657; doi:10.1371/journal.pone.0254435)
Supplement: S2 File — Spanish. (DOCX) [file pone.0254435.s002.docx]

**S2 File. CUESTIONARIO WEB**

**Proyecto de Investigación titulado “Factores de riesgo y de protección de las conductas preventivas de COVID-19”**

***Responde todas las preguntas seleccionando una respuesta que considere adecuada por pregunta, o completa la información solicitada en donde se indica.***

| 1. DATOS GENERALES  1.1. ¿Cuál es su edad (en años cumplidos)? _________  1.2. ¿Cuál es su sexo?   1. Femenino 2. Masculino   1.3. ¿Dónde vive?   1. Ciudad de México 2. Estado de México 3. Otro. Especifique________________________   1.4. ¿Qué servicios de salud utiliza habitualmente?   1. Instituciones de seguridad social (Instituto Mexicano del Seguro Social (IMSS), o Instituto de Seguridad y Servicios Sociales de los Trabajadores del Estado (ISSSTE), o Petróleos Mexicanos (PEMEX), o Instituciones de Salud de la Secretaría de la Defensa Nacional (SEDENA) 2. Instalaciones de la secretaria de Salud/Instituto de Salud para el Bienestar 3. Consulta privada/hospital privado   1.5. ¿Ud. a qué se dedica?   1. Estudiante 2. Profesional (profesional es la persona que ejerce una profesión a base de conocimiento adquirido en una universidad u otra instrucción de educación superior) 3. Profesional de salud 4. Empleado administrativo 5. Actividades del hogar 6. Comerciante 7. Trabajadores no calificados (e.j., obrero, limpieza, personal de intendencia, camarera) 8. Pensionado(a) o jubilado(a) 9. Otro. Especifique_______________________________   1.6. ¿Cuál fue el último grado escolar que terminó?   1. Ninguno 2. Primaria 3. Secundaria 4. Preparatoria o bachillerato o carrera técnica o comercial 5. Licenciatura 6. Postgrado   1.7. ¿Cuál es su estado civil?   1. Soltero 2. Casado 3. Unión libre 4. Divorciado 5. Separado 6. Viudo   1.8. ¿En la casa donde usted vive, también viven con usted niños y adolescentes menores de 18 años?  (1) Si  (2) No  1.9. ¿En la casa donde usted vive, también viven con usted adultos mayores (65 años y más)?  (1) Si  (2) No |
| --- |
| 2. ANTECEDENTES DE ENFERMEDADES  2.1. ¿Tiene alguna enfermedad crónica?  (1) Sí  (2) No -> En caso de respuesta negativa pasar a la pregunta 3.1  2.2. ¿Qué enfermedad crónica tiene? (Por favor, especifique todas las enfermedades crónicas que tiene)   \| (1). Diabetes  (2). Hipertensión arterial  (3). Enfermedad cardiovascular  (4). Enfermedad renal crónica  (5). Cáncer  (6). Obesidad  (7). Enfermedad pulmonar obstructiva crónica, o asma  (8) Otra (Especifique) \| **(1) Si** \| **(0) No** \| \| --- \| --- \| --- \|   2.3 ¿Este año Ud. tuvo la enfermedad por COVID-19?   1. Sí 2. No 3. No sé |
| 3. HÁBITOS  3.1 ¿Ud. Fuma?   1. Sí 2. No   3.2. Durante la pandemia, ¿ha hecho ejercicio físico (e.j., caminata rápida, natación, ciclismo, etc.) de manera regular (al menos 5 veces a la semana - 30 minutos por sesión) ?   1. Sí 2. No |
| 4. COVID-19  4.1. ¿Qué tan probable es que Ud. se infecte con el coronavirus (COVID-19)?   1. Nada probable 2. Poco probable 3. Más o menos probable 4. Muy probable 5. Extremadamente probable   4.2 ¿Qué tan grave considera que es la infección por coronavirus (COVID-19)?   1. Nada grave 2. Poco grave 3. Más o menos grave 4. Muy grave 5. Extremadamente grave    1. ¿Qué tanto crees que sirven las medidas de prevención de COVID-19 recomendadas por el gobierno? 6. Muchísimo 7. Mucho 8. Regular 9. Poco 10. Nada   4.4 ¿Qué tan seguro(a) esta de que Ud. tomando las medidas preventivas disminuya la probabilidad de infectarse por COVID-19?   1. Nada seguro(a) 2. Poco seguro(a) 3. Más o menos seguro(a) 4. Muy seguro(a) 5. Extremadamente seguro(a) |
| 4.5. ¿A través de qué medio recibe la información sobre la epidemia de COVID-19? (Por favor, especifique todas las fuentes que utiliza)   \| **Medio(s)** \| **(1) Si** \| **(0) No** \| \| --- \| --- \| --- \| \| (1) Televisión \|  \|  \| \| (2) Radio \|  \|  \| \| (3) Prensa (ej., periódico/revistas en formato impreso o electrónico) \|  \|  \| \| (4) Sitios de Internet \|  \|  \| \| (5) Página Web del IMSS \|  \|  \| \| (6) Página Web de la Secretaría de Salud \|  \|  \| \| (7) Correo electrónico \|  \|  \| \| (8) Redes sociales (Facebook, Twitter, WhatsApp, Instagram, YouTube y otras) \|  \|  \| \| (9) Conferencias diarias de la Secretaria de Salud sobre COVID-19 en televisión \|  \|  \| \| (10) Mensajes telefónicos \|  \|  \| \| (11) Miembros de la familia \|  \|  \| |
| 4.6 ¿Qué acciones preventivas utiliza actualmente para evitar el contagio y la propagación del COVID-19? (Por favor, especifique todas las acciones preventivas que realiza)   \|  \| **(1) Si** \| **(0) No** \| \| --- \| --- \| --- \| \| (1) Lavarse las manos con frecuencia \|  \|  \| \| (2) Usar desinfectante de manos \|  \|  \| \| (3) Usar cubrebocas fuera de su casa \|  \|  \| \| (4) Cubrir la boca al toser o estornudar en el ángulo del brazo \|  \|  \| \| (5) Evitar tocarte el rostro (ojos, nariz y boca) \|  \|  \| \| (6) Evitar contacto con personas enfermas con una enfermedad respiratoria \|  \|  \| \| (7) Desinfectar superficies con frecuencia \|  \|  \| \| (8) Mantener al menos 1.5 metros de distancia de los demás en lugares públicos \|  \|  \| \| (9) Autoevaluación del riesgo de COVID-19 utilizando aplicaciones web gubernamentales y de instituciones de salud. \|  \|  \| \| (10) No saludar de mano, ni de beso \|  \|  \| \| (11) Cambiarte y lavar la ropa después de regresar de la calle \|  \|  \| \| (12) Evitar usar transporte colectivo \|  \|  \| \| (13) Evitar acudir a lugares públicos (por ejemplo, centros comerciales, cines, restaurantes) \|  \|  \| \| (14) Evitar reunirte con grupos de más de 5 personas \|  \|  \| \| (15) Estudiar o trabajar desde casa \|  \|  \| \| (16) Evitar salir de casa mayor tiempo posible \|  \|  \| \| (17) No viajar (fuera de la ciudad, o país) \|  \|  \| \| (18) Pedirle a familiares/amigos más jóvenes que vayan a hacer compras por usted para no salir de casa \|  \|  \| \| (19) Otros (Especificar todos los faltantes)___________________ \|  \|  \| |

**5. PERCEPCIÓN DE LA SALUD (SF-12)**

| Responde todas las preguntas seleccionando una respuesta que considere adecuada por pregunta: |  |
| --- | --- |
| 5.1 En general, Ud. diría que su salud es: | \| 1 \| 2 \| 3 \| 4 \| 5 \| \| --- \| --- \| --- \| --- \| --- \| \| Excelente \| Muy buena \| Buena \| Regular \| Mala \| |
| Las siguientes preguntas se refieren a actividades o cosas que Ud. podría hacer en un día normal, Su salud actual ¿Le limita para hacer esas actividades o cosas? Si es así, ¿cuánto? | |
| 5.2 Esfuerzo moderado, como mover una mesa, empujar la aspiradora, o jugar a los bolos | \| 1 \| 2 \| 3 \| \| --- \| --- \| --- \| \| Sí, me limita mucho \| Sí, me limita poco \| No, no me limita \| |
| 5.3 Cuánto subir varios pisos por la escalera? | \| 1 \| 2 \| 3 \| \| --- \| --- \| --- \| \| Sí, me limita mucho \| Sí, me limita poco \| No, no me limita \| |
| Durante las 4 últimas semanas ¿ha tenido algún problema en su trabajo o en sus actividades cotidianas, a causa de su salud física por lo que:  5.4 hizo menos de lo que hubiera querido hacer?   1. Sí 2. No | |
| 5.5 tuvo que dejar de hacer algunas tareas en su trabajo o en sus actividades cotidianas?   1. Sí 2. No | |
| Durante las 4 últimas semanas ¿ha tenido algún problema en su trabajo o en sus actividades cotidianas, a causa de algún problema emocional como estar triste, deprimido, o nervioso por lo que:  5.6 hizo menos de lo que hubiera querido hacer?   1. Sí 2. No | |
| 5.7 No hizo su trabajo o sus actividades cotidianas tan cuidadosamente como de costumbre?   1. Sí 2. No | |
| 5.8 Durante las 4 últimas semanas, ¿Hasta qué punto el dolor le ha dificultado su trabajo habitual (¿incluido el trabajo fuera de casa y las tareas domésticas?) | \| 1 \| 2 \| 3 \| 4 \| 5 \| \| --- \| --- \| --- \| --- \| --- \| \| Nada \| Un poco \| Regular \| Bastante \| Mucho \| |
| Las preguntas que siguen se refieren a cómo se ha sentido y cómo le han sido las cosas durante las 4 últimas semanas. En cada pregunta responda cómo se ha sentido usted. Durante las 4 últimas semanas ¿cuánto tiempo | |
| \|  \| 1 \| 2 \| 3 \| 4 \| 5 \| 6 \| \| --- \| --- \| --- \| --- \| --- \| --- \| --- \| \|  \| Siempre \| Casi siempre \| Muchas veces \| Algunas veces \| Sólo alguna vez \| Nunca \| \| 5.9 …se sintió calmado y tranquilo? \|  \|  \|  \|  \|  \|  \| \| 5.10 …tuvo mucha energía? \|  \|  \|  \|  \|  \|  \| \| 5.11 …se sintió desanimado y triste? \|  \|  \|  \|  \|  \|  \| \| 5.12. Durante las 4 últimas semanas ¿con que frecuencia la salud física o los problemas emocionales le han dificultado sus actividades sociales (¿cómo visitar a los amigos o familiares? \|  \|  \|  \|  \|  \|  \| | |

| **6. ALFABETIZACIÓN EN SALUD (Dominio de prevención de enfermedades del HLS-EU-Q47)**  De las siguientes preguntas seleccione una respuesta: | Muy fácil | Fácil | Difícil | Muy difícil | No sabe |
| --- | --- | --- | --- | --- | --- |
| Preguntas | 1 | 2 | 3 | 4 | 5 |
| (6.1) ¿Qué tan fácil o difícil es para usted encontrar información para saber qué hacer con los hábitos no saludables como fumar, no hacer ejercicio físico o beber alcohol en exceso? |  |  |  |  |  |
| (6.2) ¿Qué tan fácil o difícil es para usted encontrar información sobre la manera de tratar problemas de salud mental como el estrés o la depresión? |  |  |  |  |  |
| (6.3) ¿Qué tan fácil o difícil es para usted encontrar información sobre vacunas o pruebas de detección temprana que se debería hacer (Ejemplos: la información sobre prueba de laboratorio para la detección de los niveles de azúcar en sangre o la medición de la presión arterial)? |  |  |  |  |  |
| (6.4) ¿Qué tan fácil o difícil es para usted encontrar información sobre cómo prevenir y cuidar problemas de salud como el sobrepeso, hipertensión arterial o niveles de colesterol altos? |  |  |  |  |  |
| (6.5) ¿Qué tan fácil o difícil es para usted comprender las advertencias de salud relacionadas con hábitos como fumar, no hacer ejercicio físico o beber alcohol en exceso? |  |  |  |  |  |
| (6.6) ¿Qué tan fácil o difícil es para usted entender por qué necesita vacunarse? |  |  |  |  |  |
| (6.7) ¿Qué tan fácil o difícil es para usted comprender por qué necesita hacerse pruebas de detección temprana de enfermedades o chequeos médicos? |  |  |  |  |  |
| (6.8) ¿Qué tan fácil o difícil es para usted interpretar y juzgar las advertencias de salud relacionadas con hábitos como fumar, no hacer ejercicio físico o beber alcohol en exceso? |  |  |  |  |  |
| (6.9) ¿Qué tan fácil o difícil es para usted interpretar y juzgar cuando necesita acudir al médico para hacerse una revisión médica? |  |  |  |  |  |
| (6.10) ¿Qué tan fácil o difícil es para usted interpretar y juzgar cuáles son las vacunas que puede necesitar? |  |  |  |  |  |
| (6.11) ¿Qué tan fácil o difícil es para usted interpretar y juzgar cuáles son las pruebas de detección temprana de enfermedades y las revisiones médicas que debería realizarse? |  |  |  |  |  |
| (6.12) ¿Qué tan fácil o difícil es para usted interpretar y juzgar qué tanto se puede confiar en la información sobre riesgos para la salud que aparece en los medios de comunicación (Ejemplos: información en la TV, Internet u otros medios de comunicación sobre los riesgos para la salud, como sedentarismo, consumo de tabaco, ¿etc.)? |  |  |  |  |  |
| (6.13) ¿Qué tan fácil o difícil es para usted decidir si debe ponerse la vacuna contra la influenza? |  |  |  |  |  |
| (6.14) ¿Qué tan fácil o difícil es para usted decidir cómo prevenir enfermedades gracias a los consejos que le dan la familia y los amigos? |  |  |  |  |  |
| (6.15) ¿Qué tan fácil o difícil es para usted decidir cómo prevenir enfermedades gracias a la información que proporcionan los medios de comunicación? |  |  |  |  |  |
